# Research on Taproots Identification Technology in *Panax notoginseng* Quality Intelligent Management System

**DOI:** 10.1155/2021/8292535

**Published:** 2021-09-16

**Authors:** Mingfang Chen, Zhongping Chen, Xiuming Cui, Yongxia Zhang, Sen Wang

**Affiliations:** ^1^School of Mechanical and Electrical Engineering, Kunming University of Science and Technology, Kunming 650500, China; ^2^Key Laboratory of Sustainable Utilization of Panax Notoginseng Resources in Yunnan Province, Kunming 650500, China

## Abstract

In the *Panax notoginseng* quality intelligent management system, the big roots and fibrous roots cannot be cut automatically because the machine cannot distinguish the taproot, big roots, and fibrous roots of *Panax notoginseng*, resulting in the automatic cutting mechanism unable to obtain the control trajectory coordinate reference of the tool feed. To solve this problem, this paper proposes a visual optimal network model detection method, which uses the image detection method of marking anchor frames to improve the detection accuracy. A variety of deep learning network models are modified by the TensorFlow framework, and the best training model is optimized by comparing the results of training, testing, and verification data. This model is used to automatically identify the taproots and provide the control trajectory coordinate reference for the actuator that cuts big roots and fibrous roots automatically. The experimental results show that the optimal network model studied in this paper is effective and accurate in identifying the taproots of *Panax notoginseng*.

## 1. Introduction

**Figure 1:**
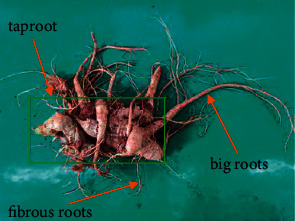
*Panax notoginseng*.

The *Panax notoginseng* industry is an important component of Yunnan province in China. The Yunnan *Panax notoginseng* processing plant needs to process a big amount of *Panax notoginseng* raw materials in harvest season. These raw materials will be powdered or sliced to prepare them for use in subsequent pharmaceuticals. However, there is an important and critical step in the process of making *Panax notoginseng* powder or slicing is to cut big roots and fibrous roots. The harvested *Panax notoginseng* is divided into three parts: the taproot, the big roots, and the fibrous roots; *Panax notoginseng* is shown in Figure 1. Although the effective ingredients contained in the three parts are the same, the saponin content of each part is quite different. Therefore, adding a big proportion of big roots and fibrous roots will reduce the effective content of saponin and affect the subsequent pharmaceuticals. At present, the big roots and fibrous roots of *Panax notoginseng* are cut by manual visual inspection. Due to the different shapes and sizes of *Panax notoginseng*, human visual inspection will have different results because everyone has different criteria for judging the big roots and fibrous roots of *Panax notoginseng*, so the standards for cutting are different. Because long hours of manual work will cause visual fatigue, there will be different judgment standards at different times. These different judgment standards will cause differences in shearing, which will affect the subsequent pharmaceuticals made form, *Panax notoginseng*.

Target detection aims to find all objects of interest through the computer, including classifying the objects and determining the location of the objects [1]. It could not only effectively reduce the interference of human factors but also realize stable identification and high-precision detection. Therefore, target detection is widely used in industry and agriculture [2]. Common target detection is divided into traditional target detection and target detection based on deep learning [3].

Traditional target detection mainly uses artificial selection of features to detect objects [4]. Detection is usually divided into three stages. First, a certain part of the image is determined as a candidate area through a multiscale sliding window, then the features of the candidate area are extracted by the constructed features, and the classifier is used for classification [5–8]. Traditional target detection could better realize target recognition in which the image was in specific scenes and fewer categories [9]. However, if the external environment, such as the lighting changes, it is difficult to achieve a good detection effect, since it is easy to miss or incorrectly detect. At the same time, traditional target detection will also generate a large number of candidate boxes, which increases the computational overhead, and the accuracy and speed of detection cannot meet the requirements of practical industrial applications [10, 11].

Viola et al. [12, 13] used the Viola Jones detector to achieve real-time face detection for the first time by a direct detection method without any constraints at the time, but it required more computational ability than available at that time. Dalal and Triggs [14] used a directional gradient histogram feature descriptor that had a superior recognition ability for pedestrian detection, However, this algorithm is prone to missed detection when dealing with occlusion, large posture changes, and target angle change [15]. Felzenszwalb et al. [16] proposed a model based on variable parts to detect the target object through the ideas of an improved histogram of gradient (HOG) feature and support vector machine (SVM) classifier. This detection method has a certain robustness for deformed targets, but the method has poor stability for large-scale rotating targets and requires an artificial design of different excitation templates for detection.

Target detection based on deep learning has achieved great success in the field of detection [17]. Compared with traditional target detection, this algorithm based on deep learning could not only use fewer data sets to obtain high-precision detection results but also greatly improve the speed of target detection [18]. Therefore, it is widely used in industry and agriculture.

Aiming at the fact that currently the big roots and fibrous roots of *Panax notoginseng* are cut manually, this article proposed a visual inspection method for developing an optimal model of identifying *Panax notoginseng* taproots. This method could improve the accuracy and stably identify the taproots of *Panax notoginseng* and be prepared for the subsequent cutting of the big roots and fibrous roots of *Panax notoginseng* by machines. To improve the stability and reliability of target detection and to meet the need for rapid detection in industry, this paper uses the bounding box method to mark the anchor frame to detect the target. To reduce the number of training samples, this article applies LabelImg mapping software to mark the target features of limited samples and rely on the TensorFlow framework to modify a variety of different deep learning network models. By comparing the training, testing, and verification performance of different network models, this article selects the optimal network model to detect the taproots of *Panax notoginseng*.

## 2. Detection of the Taproot Target of *Panax notoginseng*

**Figure 2:**
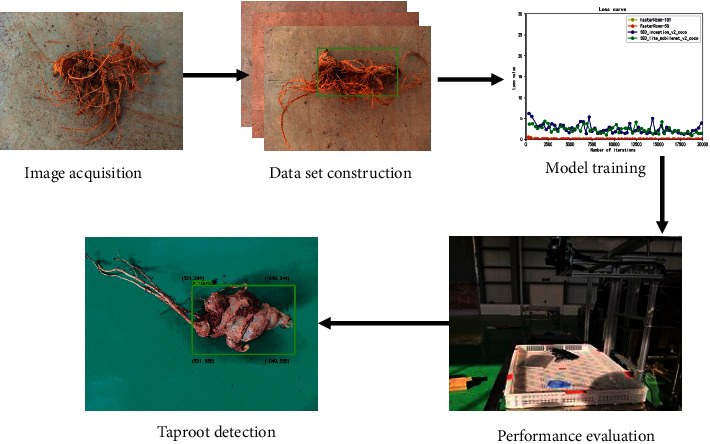
Determination of the taproot of *Panax notoginseng*.

Visual detection of the *Panax notoginseng* taproots involved five steps: image acquisition of *Panax notoginseng*, database construction, network model training, model performance comparison, and *Panax notoginseng* taproot detection. The specific implementation process is shown in Figure 2.

### 2.1. Image Acquisition of *Panax notoginseng*

**Figure 3:**
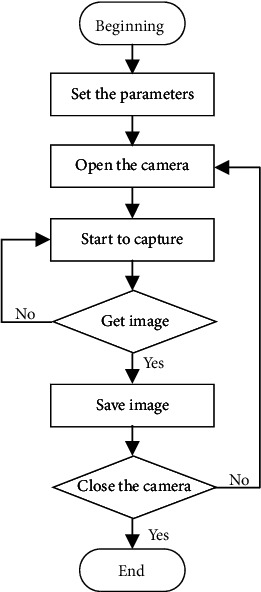
Image acquisition flowchart.

The taproot detection of *Panax notoginseng* requires the collection of *Panax notoginseng* images. The acquisition tool used was CMOS industrial camera (Basler ace-acA2500-14gc, F1.4-F16), which has a higher acquisition frequency and acquisition speed. The use of a vision and motion module in LabVIEW to collect images of *Panax notoginsen*g can fully meet the requirements of industrial collection [19]. The flowchart of image acquisition is shown in Figure 3. Through the collection results, this paper found that although the use of higher resolution provided a wider field of view in the image, it required a larger storage space and took more time for image collection and processing. The low image resolution reduced the field of view of the image, but it saved the storage space of the image and reduced the image collection and processing time. Because the detection of the taproot of *Panax notoginseng* was a large target detection, the recognition of *Panax notoginseng* taproots does not require higher acquisition speed and higher resolution in actual industrial applications, so this article sets the acquisition resolution of the industrial camera at 1156 × 868 and sets the rate of image acquisition to 1 frame/sec.

### 2.2. Dataset Construction

**Figure 4:**
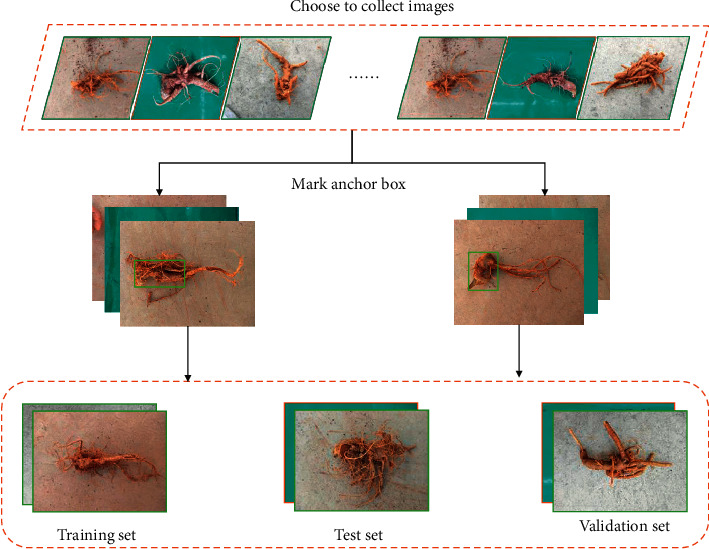
Dataset construction.

It was necessary to construct a dataset of *Panax notoginseng* images before using detection on the taproot. In this paper, we selected *Panax notoginseng* images that contained only big root and fibrous root images from the collected images to construct the entire image dataset. The taproots of *Panax notoginseng* images have different directions, positions, shapes, and backgrounds. The big root and fibrous root images also had different shapes and backgrounds. This article used a random sampling method to divide the *Panax notoginseng* image dataset into a training set, test set, and validation set according to a certain proportion so that the samples were more random and extensive. This article also used the LabelImg plotting tool to anchor the taproot in the training set and test set of *Panax notoginseng*. We integrated the files generated after the anchor frame with the corresponding image files to jointly construct the dataset. The process of constructing the dataset is shown in Figure 4.

### 2.3. Network Model Training

**Figure 5:**
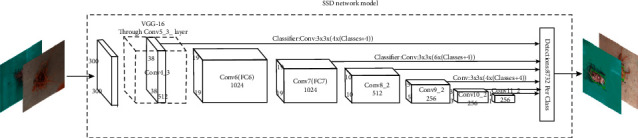
SSD network model skeleton.

At present, the big roots and fibrous roots of *Panax notoginseng* are still mainly done by manual cutting. The shape of the roots are complicated, different workers have different standards for judging big roots during cutting, and the same workers also have different standards for cutting in different time periods during the day. Target detection based on deep learning can solve the problem of inconsistent manual detection standards, and at the same time, it can avoid the situation of incorrect detection or missed detection of traditional target detection in a complex background with multiple features. Considering the practical applications of engineering, this article used the bounding box method to mark the anchor box to identify the taproot of *Panax notoginseng*, wrote the entire deep learning model framework in Python, and used the constructed *Panax notoginseng* dataset. A public deep learning model downloaded from the Internet was used as a pretraining model, and the taproot recognition of *Panax notoginseng* was realized by modifying the parameters of the training model. The selected network model includes single-stage SSD [20] and two-stage faster-RCNN [21] as the network pretraining model. Under the TensorFlow framework, different network models were trained and tested, the average test accuracy between different optimal models was verified, and the optimal network that meets the taproot detection requirements was selected. The verification formula used in this article is shown in formulas (1) and (2), and the SSD model skeleton [22] used to train detection of the *Panax notoginseng* taproot is shown in Figure 5, where *N* is the total number of sheets in the training set and test set, *N* ∈ (1, 2,…, *N*), *P*_*N*_ is the detection accuracy of the model, AVPE is the average detection accuracy of the model, TP_*N*_ is the number of *Panax notoginseng* taproots correctly detected, and FP_*N*_ is the number of background detections as the taproot of *Panax notoginseng*:

(1) $$\begin{array}{c} P_{N}=\frac{{\text{TP}}_{N}}{{\text{TP}}_{N}+{\text{FP}}_{N}}, \end{array}$$

(2) $$\begin{array}{c} \text{AVPE}=\frac{1}{N}\overset{N}{\underset{1}{\sum }}\left(P_{1},P_{2},,,P_{N}\right). \end{array}$$

### 2.4. Model Performance Comparison

This paper used different deep learning network models to detect the taproot of *Panax notoginseng*. However, there are obvious differences in the detection effects of different network models, and the average detection accuracy could not effectively select the optimal network model. To better realize the comparison of various network models and select the optimal network model, this paper proposed a comprehensive performance verification formula that combines average accuracy, accuracy, missed detection rate, and false detection rate for deep learning model performance evaluation and comparison. The verification formula is shown in (6), where *H*_*p*_ is the number of the taproots of *Panax notoginseng* predicted by the deep learning model, *H*_*t*_ is the sample size of the real *Panax notoginseng* taproot, *H*_*l*_ is the number of missed detections of the taproot of *Panax notoginseng*, and *H*_*f*_ is the number of errors during the detection. In this paper, the determined deep learning model was verified, and the performance of different models was evaluated on the validation results until the optimal network model was selected:

(3) $$\begin{array}{c} P_{a}=\frac{H_{p}}{H_{t}}, \end{array}$$

(4) $$\begin{array}{c} P_{l}=\frac{H_{l}}{H_{t}}, \end{array}$$

(5) $$\begin{array}{c} P_{f}=\frac{H_{f}}{H_{t}}, \end{array}$$

(6) $$\begin{array}{c} C_{\text{PE}}=\frac{1}{N}\overset{N}{\underset{1}{\sum }}\left(\text{mean}\left(\text{AVPE}+P_{a}\right)-P_{l}-P_{f}\right). \end{array}$$

### 2.5. The Taproot Detection of *Panax notoginseng*

**Figure 6:**
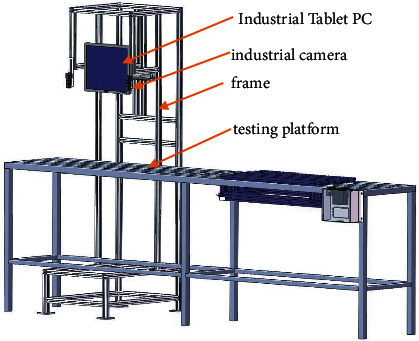
Hardware 3D diagram of taproot detection.

**Figure 7:**
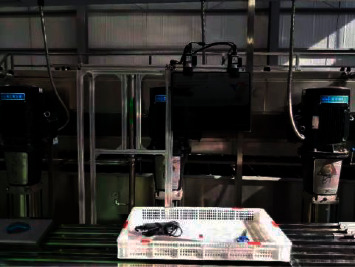
Physical detection platform.

**Figure 8:**
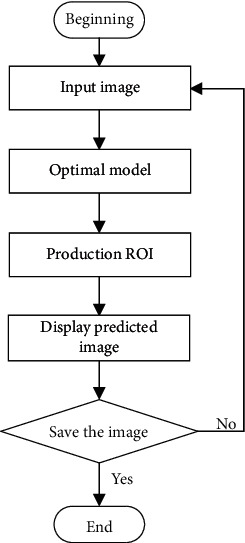
Flowchart of taproot detection of *Panax notoginseng*.

**Figure 9:**
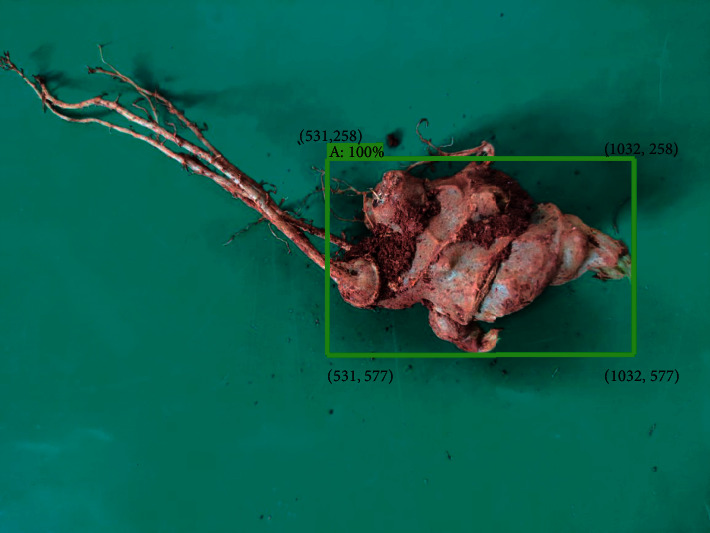
An example of taproot detection of *Panax notoginseng*.

*Panax notoginseng* taproot detection was to perform image detection on the verification set according to the optimal model trained in deep learning. The hardware 3D diagram of detection is shown in Figure 6. The physical platform diagram is shown in Figure 7. The hardware mainly includes industrial cameras, industrial tablet computers, racks, and test platforms. Among them, the test platform was used, and the industrial tablet computer was used to embed the optimal network model to realize the detection and result display of the images collected by the industrial camera. The detection flowchart is shown in Figure 8, and the detection result is shown in Figure 9.

## 3. Recognition Results and Analysis of the Taproot of *Panax Notoginseng*

### 3.1. Experimental Environment and Configuration

For the effectiveness of the visual inspection of the taproot of *Panax notoginseng*, 175 images with multiple backgrounds and multiple shapes containing the taproot and 25 images with only big roots and fibrous roots were selected from the 1685 collected images. The 200 images were divided into a training set, test set, and validation set at a ratio of 7 : 2:1 by random sampling. The experimental platform environment is 2(Intel (R) Core(TM)i7-9700F CPU@3.00 GHz, GPU GeForce RTX 2070S, 32 GB memory, Windows7-64bit. The deep learning network model used two frameworks: SSD and Faster-RCNN under TensorFlow. The 4 network models included SSD_inception_v2_coco, SSD _ Lite _ mobilenet_v2_coco, Faster-RCNN-Resnet50, and Faster-RCNN-Resnet101. We modified and trained the pretraining models of these 4 network models; the results of training used loss, AVPE, CPE, Pa, Pl, and Pf to compare the qualitative and quantitative analysis of the entire model.

### 3.2. Model Training and Verification

**Figure 10:**
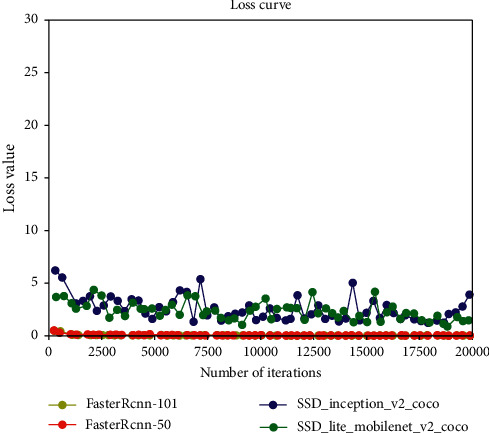
Four model loss function curves.

**Figure 11:**
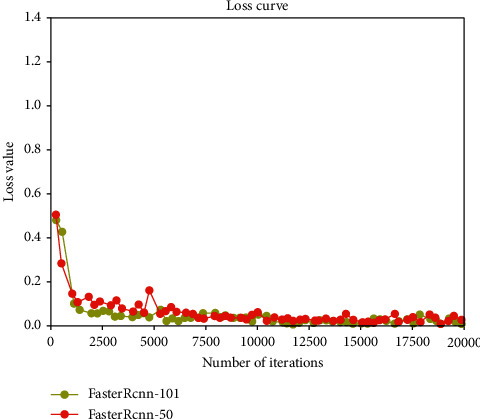
Faster-RCNN loss function curve.

**Figure 12:**
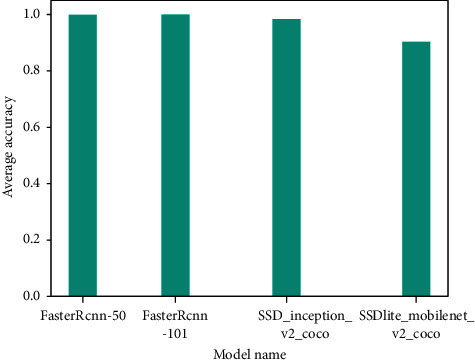
Average accuracy of the model.

The results of training and verification of the four network models were batchSize = 4, learningRate = 0.0001, the number of iterations was 20,000, and the other parameters were defaulted. The generated iteration number and loss value curve are shown in Figure 10. It can be seen from the figure that the loss value of SSD_inception_v2_coco was the largest at the beginning, and the loss values of RCNN-Resnet101 and Faster-RCNN-Resnet50 were the smallest. However, after 1000 iterations of SSD_ inception_ v2_coco, the loss value decreased, but the model loss function curve was in a state of nonconvergence. At the same time, the loss function curve of the SSD _ Lite _ mobilenet _ v2 _coco model was also in a state of nonconvergence, and the loss functions of SSD _ Lite _ mobilenet _ v2 _ coco and SSD_ inception _v2_coco were always higher than the function value of Faster-RCNN. The two model networks of Faster-RCNN had relatively small loss values at the beginning, as shown in Figure 11. However, compared with Faster-RCNN-Resnet50, the loss value of Faster-RCNN-Resnet101 was generally lower than that of Faster-RCNN-Resnet50. Although the loss value of Faster-RCNN-Resnet50 was lower than Faster-RCNN-Resnet101 when the number of iterations was 18000 times, the loss value of Faster-RCNN-Resnet101 was lower afterward. Therefore, compared with the four network models, Faster-RCNN-Resnet101 had a better modification effect. The comparison and verification of the optimal model among the four training models is shown in Figure 12. When the number of iterations was 19,811, the Faster-RCNN-Resnet101 model could detect the optimal verification result with an average detection rate of 99.99% for the taproot. The Faster-RCNN-Resnet50 model could detect the taproot with an average detection accuracy of 99.90% when the number of iterations was 17,795. When the number of iterations of SSD _ Lite _ mobilenet_v2_coco was 17987, the average detection accuracy of the taproot of the optimal model was 98.36%. The optimal model of SSD _ inception _v2 _coco had a poor average detection rate for the taproot, which was only 90.25%. The comprehensive results show that using the *Panax notoginseng* dataset, Faster-RCNN-Resnet101 was more suitable for target detection than the other models [23].

### 3.3. Model Testing and Comparison

**Figure 13:**
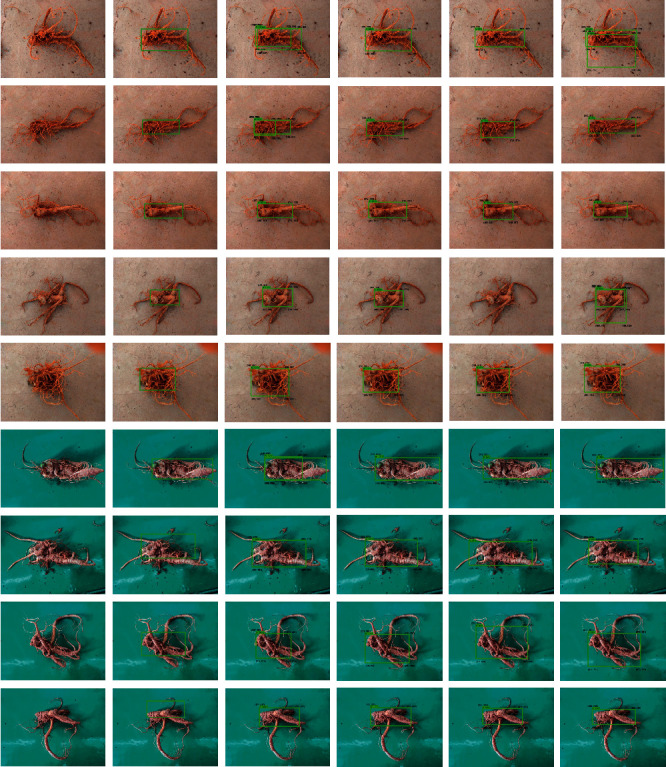
Comparison results of partial qualitative results of the validation of the four optimal models. From left to right, raw image, GT, Faster-RCNN-Resnet50, Faster-RCNN-Resnet101, SSD_INCPTION_V2_COCO, and SSD_LITE_MOBILEET_V2_COCO.

**Figure 14:**
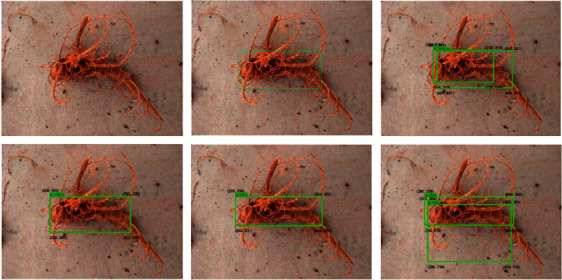
Comparison result of row 1 in Figure 13.

**Figure 15:**
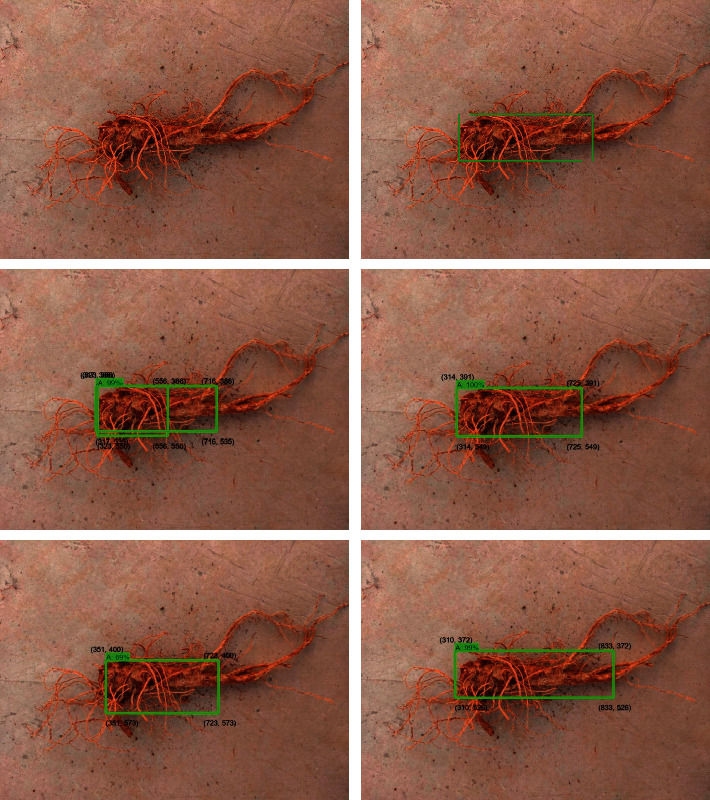
Confidence comparison of row 2 in Figure 13.

To effectively verify the universality of the four deep learning models for the detection of the taproot of *Panax notoginseng*, in this paper, 18 images from the *Panax notoginseng* validation set were used to compare and analyze the performance of the four optimal performance models. Part of the qualitative analysis is shown in Figure 13. By comparing and analyzing the results, it could be seen that the four network models of SSD_inception_v2_coco, SSD _ Lite _ mobilenet _ v2 _coco, Faster-RCNN-Resnet50, and Faster-RCNN-Resnet101 can detect the taproot of *Panax notoginseng*, but the detection of SSD_inception_v2_coco was unstable, and the detection of some complex multiroot images would be missed. We listed all of the model detection results in the first row in Figure 13 and observed the categories and detection frame positions of the *Panax notoginseng* taproots by the four models. The enumeration diagram is shown in Figure 14. Through observation, it was found that Faster-RCNN-Resnet101 can stably detect the taproot target. The SSD_Lite_mobilenet_v2_coco and Faster-RCNN-Resnet50 models can also detect all taproot images. However, for complex *Panax notoginseng* images, there would be a problem of detecting multiple frames. Further qualitative comparison of some of the verification images in the *Panax notoginseng* verification set was carried out to achieve the accuracy comparison of the bounding box.  Figure 15 shows the comparison result of a certain area randomly selected in this paper. The frame selection accuracy of the four models would have certain differences. Taking the second row in Figure 13 as an example, Faster-RCNN-Resnet101 could detect surrounding targets other than the taproot of *Panax notoginseng*, and the detection confidence was 100%. Faster-RCNN-Resnet50 could also detect the taproot target, but it could not be detected correctly, and the detection accuracy rate was also lower than that of Faster-RCNN-Resnet101. The two models SSD_Lite_mobilenet_v2_coco and SSD _ inception _ v2_coco could be detected correctly, but the detection confidence was lower than that of Faster-RCNN-Resnet101. The quantitative comparison results in Table 1 also show that Faster-RCNN-Resnet101 had a stable and good detection effect on the taproot of *Panax notoginseng*. Therefore, by comparing the results, it is concluded that Faster-RCNN-Resnet101 was more suitable for the visual inspection of the taproot of *Panax notoginseng*.

## 4. Conclusion

To avoid the defects of manual visual inspection and realize the taproot target recognition of *Panax notoginseng*. This article constructed an optimal model based on deep learning to detect the taproot target, relying on TensorFlow various deep learning network models for modification and training, and used the proposed optimization strategy to compare the training, testing, and verification of different network models to select the optimal network model, realizing the detection of the taproot target of *Panax notoginseng*. The experimental results showed that, by modifying and training different network models and selecting the optimal network model, the detection efficiency and accuracy of the target could be effectively improved, and the detection accuracy of the optimal model could reach 99.99%. However, this article does not give the identified taproot position parameters to the controller. Therefore, in future work, we can continue to modify the parameters of the optimal network model and transmit the taproot coordinates to the controller so that the actuator can accurately cut the big roots and fibrous roots of *Panax notoginseng*.

## Figures and Tables

**Table 1:** CPE comparison results of the four optimal model validations.

Model	*H*_*t*_	*H*_*p*_	*H*_*l*_	*H*_*f*_	*P*_*a*_ (%)	*P*_*l*_ (%)	*P*_*f*_ (%)	AVPE (%)	*C*_PE_ (%)
SSD_inception_v2_coco	18	17	1	0	94.44	5.56	0	90.25	89.57
SSD_Lite_mobilenet_v2_coco	18	16	0	2	88.89	0	11.11	98.36	88.07
Faster-RCNN-Resnet50	18	15	0	3	83.33	0	16.67	99.90	83.28
Faster-RCNN-Resnet101	18	18	0	0	100	0	0	99.99	99.99

## Data Availability

The datasets used to support the findings of the study are available from the corresponding author upon request.
